# Supplementary material for: Controllable Synthesis and Growth Mechanism of Interlayer-Coupled Multilayer Graphene
Source: Nanomaterials (Basel). 2023 Sep 25;13(19):2634. doi: 10.3390/nano13192634 (PMC10574119; doi:10.3390/nano13192634)
Supplement: Supplementary file 1 [file nanomaterials-13-02634-s001.zip › nanomaterials-2606148-supplementary.pdf]

Supporting Information

Controllable Synthesis and Growth Mechanism of Interlayer Coupled Multilayer Graphene

Xudong Xue [1,2], Mengya Liu [1,2], Xiahong Zhou [1,3], Shan Liu [1,3], Liping Wang [2] and Gui Yu [1,3,*]

[1] Beijing National Laboratory for Molecular Sciences, CAS Research/Education Center for Excellence in Molecular Sciences, Institute of Chemistry, Chinese Academy of Sciences, Beijing 100190, China; xuexudong@hebeu.edu.cn (X.X.); b20200192@xs.ustb.edu.cn (M.L.); zhouxiahong@iccas.ac.cn (X.Z.); liushan@iccas.ac.cn (S.L.)

[2] School of Materials Science and Engineering, University of Science and Technology Beijing, Beijing 100083, China; lpwang@mater.ustb.edu.cn

[3] School of Chemical Sciences, University of Chinese Academy of Sciences, Beijing 100049, China

* Correspondence: yugui@iccas.ac.cn

Characterizations: The graphene samples underwent systematic characterization using various techniques. Scanning Electron Microscopy (SEM) was performed using a Hitachi S-4800 instrument operating at 5 kV. Optical microscopy was conducted by using an Olympus BX51 microscope. Raman spectroscopy was carried out by using a Renishaw Invia plus spectrometer with a 532 nm laser for excitation. Transmission Electron Microscopy (TEM) analysis was performed by using a JEOL JEM-2100F instrument operating at 200 kV.

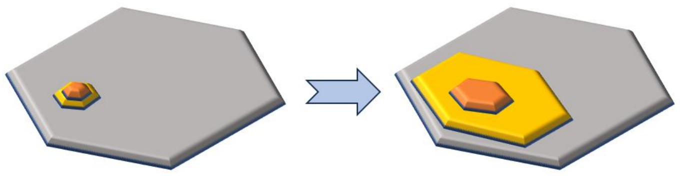

Figure S1. Growth diagram of interlayer coupling multi-layer graphene.

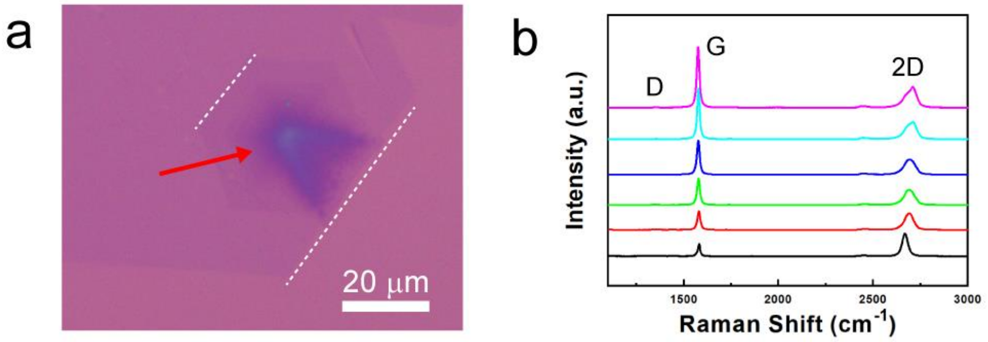

Figure S2. (**a**) Optical photograph of interlayer-coupled multilayer graphene transferred onto the SiO_2/Si surface, and (**b**) its corresponding Raman spectrum.

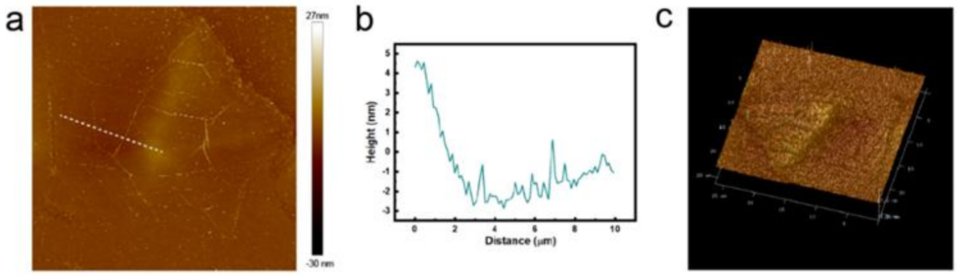

Figure S3. AFM characterization of interlayer-coupled multilayer graphene. (**a**) AFM image of interlayer-coupled multilayer graphene. (**b**) Height profile along the white dashed line in figure (**a**). (**c**) Three-dimensional AFM image of interlayer-coupled multilayer graphene.

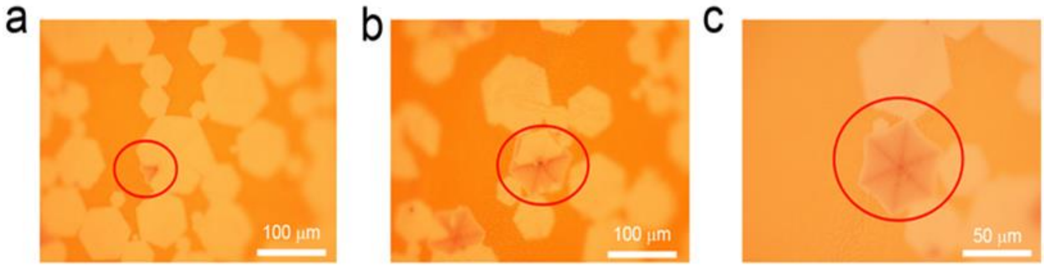

Figure S4. (**a–c**) Optical photographs of interlayer-coupled multilayer graphene with different morphologies. 500 Ar sccm, 100 sccm H_2 and 0.8sccm CH_4.

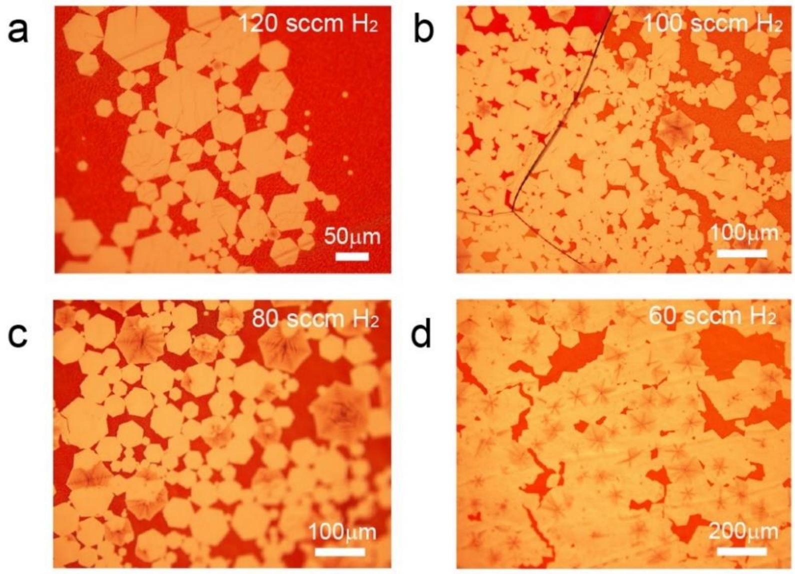

Figure S5. Optical photographs of interlayer-coupled multilayer graphene grown on liquid copper under different hydrogen gas flow rates. 500 Ar sccm, 0.8sccm CH_4 and (**a**) 120 sccm H_2, (**b**) 100 sccm H_2, (**c**) 80 sccm H_2, (**d**) 60 sccm H_2, respectively.

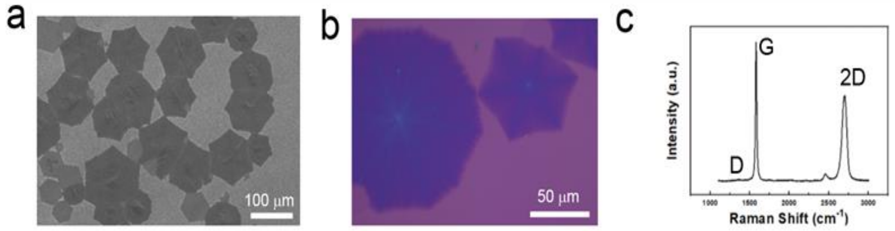

Figure S6. Morphology and structural characterization of umbrella-like multilayer graphene, (**a**) SEM image. (**b**) Optical photograph of multilayer graphene transferred onto SiO_2/Si. (**c**) corresponding Raman spectrum of the multilayer graphene in figure (**b**).

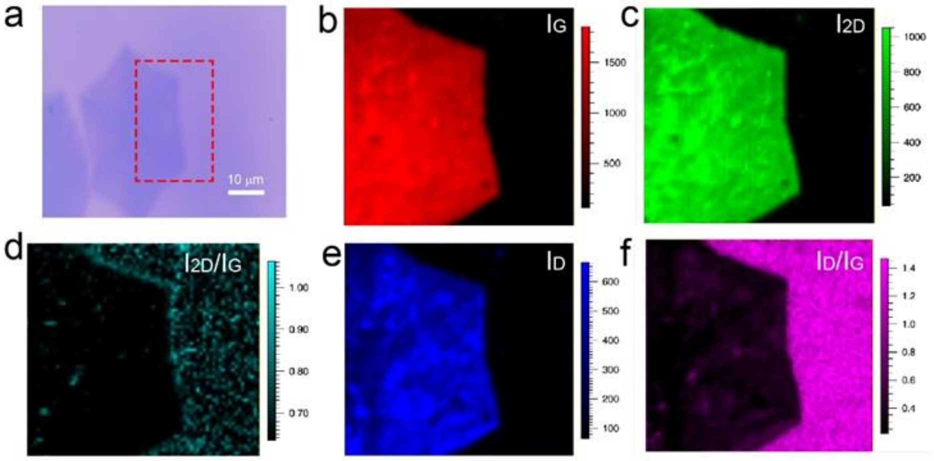

Figure S7. (**a**) Optical image of umbrella-like multilayer graphene, (**b**–**f**) Raman mapping spectra of umbrella-like multilayer graphene.

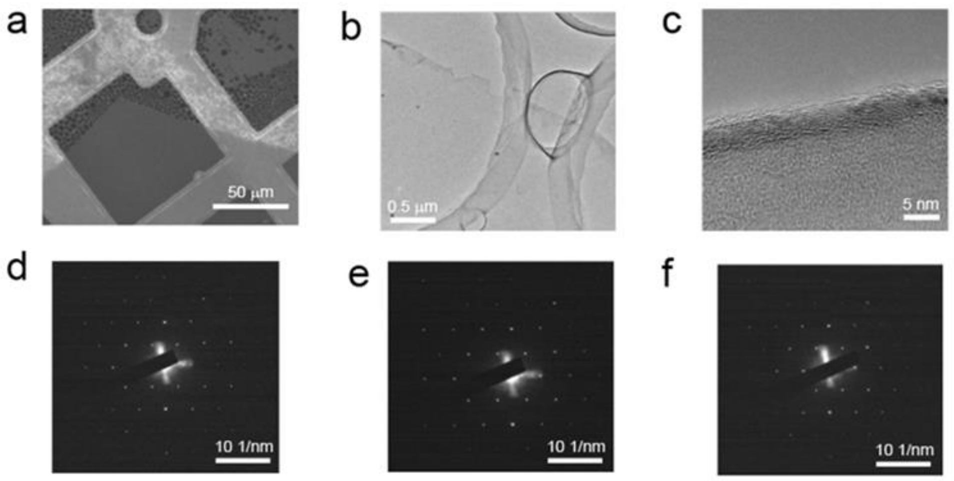

Figure S8. (**a**) SEM image of umbrella-like interlayer-coupled multilayer graphene transferred onto a microgrid, (**b**) Low-magnification TEM image of umbrella-like interlayer-coupled multilayer graphene, (**c**) High-magnification TEM image of umbrella-like interlayer-coupled multilayer graphene, (**d**–**f**) SEAD patterns of umbrella-like interlayer-coupled multilayer graphene at different locations.

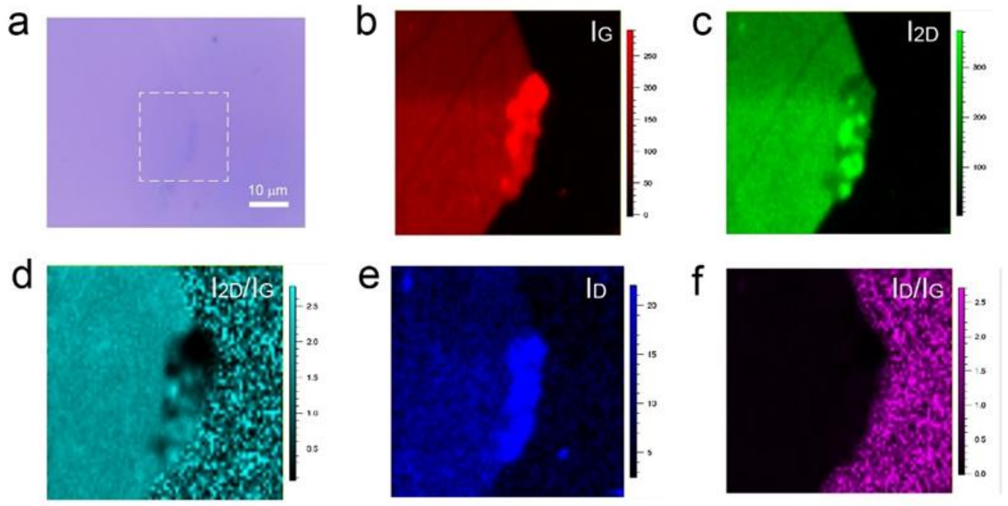

Figure S9. (**a**) Optical photograph of the sample where carbon species start to accumulate at the edges of graphene domains, (**b**–**d**) Raman mapping spectra at the position indicated by the white dashed box in Figure (**a**).

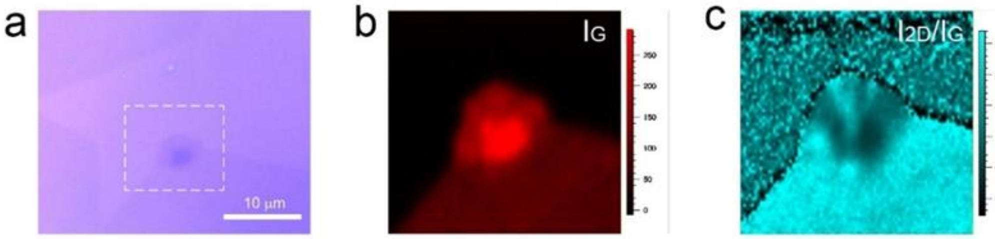

Figure S10. (**a**) Optical photograph of the sample where carbon species continue to accumulate at the edges of graphene domains, (**b**,**c**) Raman imaging spectra of the G peak and I_{2D}/I_G ratio at the position indicated by the white dashed box in Figure (**a**).

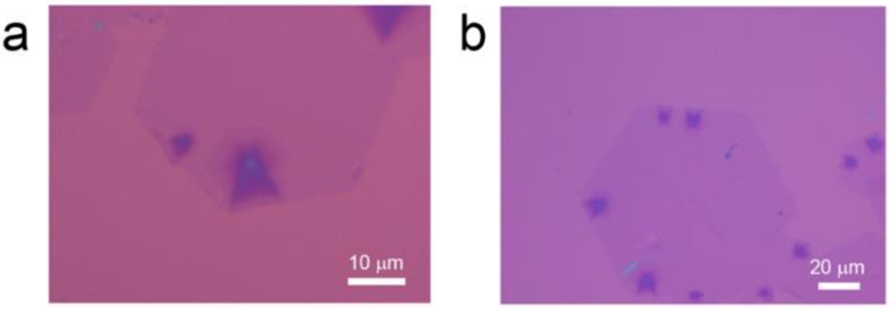

Figure S11. (**a**,**b**) Optical photographs of interlayer-coupled multilayer graphene with different morphologies transferred onto SiO_2/Si substrate.

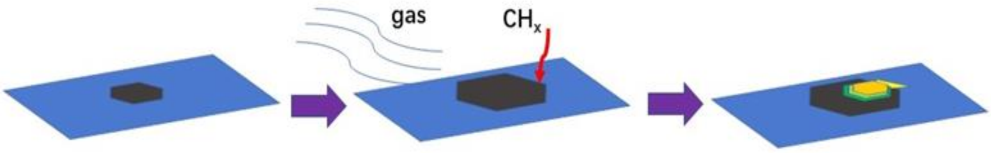

Figure S12. Schematic illustration of the growth process of winged-interlayer-coupled multilayer graphene.
